# Waterpipe tobacco smoking: A reality or hidden iceberg for Iranian women

**DOI:** 10.15171/hpp.2018.35

**Published:** 2018-10-27

**Authors:** Mohammad Ebrahimi Kalan, Ziyad Ben Taleb

**Affiliations:** Department of Epidemiology, Robert Stempel College of Public Health, Florida International University, Florida, USA

## Dear Editor,

Cigarette smoking is the leading cause of preventable death and remains the most common form of consumed tobacco, worldwide.^1^ An ancient tobacco smoking method that is regaining popularity is the waterpipe (also known as qalyan, shisha, hookah, and narghile).^2,3^ According to the World Health Organization (WHO), although global tobacco smoking prevalence rates among women are currently lower than men, they are expected to rise in many developing countries.^4^ Globally, around 10 million women died from tobacco use between 1950 and 2000, however, it is estimated that tobacco-attributable deaths among women could be potentially more than double by 2030.^5^ Across the Middle East, waterpipe tobacco smoking (WTS) seems to attract mostly young individuals with its sweet taste, delicate flavor, and most importantly, declining social disapproval, especially for women.^4,6^ In a cohort study of adolescents in Lebanon, women represented 53.8% of waterpipe smokers compared to 8.3% of cigarette smokers, reflecting the social acceptability of WTS among women in Middle Eastern contexts.^7^ The trend of WTS is increasing in Iran and has become one of the most popular leisure activities for Iranian women.^8,9^ Previous research showed that, especially in the Middle East, young women seem more likely to choose waterpipe over cigarettes and this behavior is more common despite its evidence-based detrimental health effects on this population.^8-10^ The escalating popularity of WTS has prompted Iranian medical practitioners to warn of the dire consequences for the health of their country’s youth.^11^ A most recent cross-sectional study in Iran reported that the home environment was the place where most of the participants initiated waterpipe smoking for the first time.^12^ Furthermore, a qualitative study among Iranians showed that women are susceptible to initiate WTS in three ways: (*a*) women’s involvement in the preparation of waterpipe and the frequent observation of individuals who smoke a waterpipe at home can be effective in WTS initiation among young women; (*b*) the husband of a young woman has a significant role in the initiation of WTS; (*c*) when parents invite children to smoke a waterpipe at home, in order to protect them against public disapproval, the mother may initiate WTS activity.^13^ Collectively, these individual and social features unique to WTS can influence its initiation and current use among women.

A time trend study in Ardebil, a province in northwest of Iran, among medical students presented 75% increase (44.1% to 77.1%) in flavored WTS among women in 2014 compare to 2009, while this increase was only 14.4% (65.6% to 75.0%) among men.^14^ Another study among Iranian health science students demonstrated that the women were smoking waterpipes almost as frequently as men (48% versus 52%, respectively).^15^

As shown in Table 1, it can be easily observed that the different studies have reported different prevalence rates among Iranian men and women. The smoking prevalence derived from these studies are based on the same definition of waterpipe smoking (current/or lifetime waterpipe smokers). Current WTS was defined as smoking at least once a month, while lifetime WTS was defined as smoking at least once in a life.^15^

A number of programs have been initiated to decrease the rate of waterpipe usage among the Iranian population.^24^ Yet, health policy makers are facing WTS as the most common method of tobacco smoking among young women.^9,13^ For example, the prevalence of waterpipe use among Iranian women has previously been reported to be between 6% and 8% in two studies in Iran.^8,25^ Despite these reports, WTS is considered as an emerging public health issue for Iranian women, with more younger women joining this population.^9^ On one hand, due to the social and cultural restrictions for Iranian women to smoke a waterpipe in public places (e.g. a cafe, lounge, restaurant, etc) compared to men, many young women are smoking this tobacco product at home, social gatherings, or in concealed places. Smoking with family members and a group of friends and sharing the same waterpipe are typical features of WTS.^26^ A cultural irony is that Iranian families do not object to WTS habits of women and seem to condone their behavior compare to conventional cigarette smoking. Conversely, women have more difficulty quitting WTS than men due to their responses to nicotine dependence, a lack of social support, fear of weight gain, depression, and hormones.^23,27,28^ Therefore, it is crucial that the public health providers develop and implement educational and prevention programs, as well as culturally targeted interventions focused mostly on families, and intensive group based smoking cessation treatment among women.^13,29,30^ For example, programs may involve young women in community and school-based tobacco prevention activities, and connect them with cessation services to enhance client education and access to insurance coverage for nicotine replacement medications. Additionally, replacing waterpipe smoking with another family activity can diminish the spread of WTS among Iranian families as can considering prevention as a high priority for socio-economically disadvantaged families, and introducing the courses at schools to increase awareness of health risks and side effects of WTS.^4,9,13,23,30,31-33^ Also, the lack of evidence-based studies for developing health warning labels is another challenge in preventing WTS among youth.

We believe that WTS rates among Iranian women only represent the tip of the iceberg because either many women did not report their WTS habits or not enough nationwide studies have been done among this population. Evidence-based tobacco control strategies should be implemented to limit the growth in women’s WTS rates. Also, it is important to identify the behaviors and social norms leading to the increase in WTS among Iranian women so that steps can be taken to identify strategies to curb WTS among this vulnerable population.

**Table 1:** Trend of prevalence (%) of lifetime and current waterpipe smoking among Iranian population

**Nationwide/City**	**Year**	**Sample size**	**Men**		**Women**		**Total**		**Reference**
			**Lifetime**	**Current**	**Lifetime**	**Current**	**Lifetime**	**Current**	
Tehran	2007	296^a^	-	52.0	-	48.0	-	51.0	^15^
National Survey	2007	5287^b^	-	3.5	-	1.9	-	2.7	^16^
Tehran	2010	1201^b^	51.4	34.8	38.9	21.4	45.1	28.0	^17^
Kerman	2011	682^c^	-	25.9	-	12.6	18.8	27.0	^18^
Tehran	2012	1359^d^	-	-	37.3	6.3	-	-	^19^
Tabriz	2013	456^e^	-	-	-	-	-	82.0	^20^
Tehran	2013-2014	1830^b^	-	24.2	-	11.3	-	17.6	^21^
Mashhad	2014	673^b^	-	6.5	-	11.3	-	8.6	^22^
Kerman	2016	1090^a^	56.9	36.9	30.4	20.6	43.8	28.8	^23^

^a^ University students; ^b^ General population; ^c^ Adolescent; ^d^ General population (Only women); ^e^ Waterpipe smokers.

## Ethical approval

Not Applicable.

## Competing interests

The authors declare that they have no competing interests.

## Authors’ contributions

MEK and ZBT contributed substantively to conceptualizing, writing, and revising this letter.

## Acknowledgements

The authors would like to thank Donna Davis, Rochelle Parrino and Makella Coudray for their assistance with this letter.
